# Composition of Intracellular and Cell Wall-Bound Phlorotannin Fractions in Fucoid Algae Indicates Specific Functions of These Metabolites Dependent on the Chemical Structure

**DOI:** 10.3390/metabo10090369

**Published:** 2020-09-11

**Authors:** Claudia Birkemeyer, Valeriya Lemesheva, Susan Billig, Elena Tarakhovskaya

**Affiliations:** 1 Faculty of Chemistry and Mineralogy, University of Leipzig, 04103 Leipzig, Germany; billig@uni-leipzig.de; 2 Department of Plant Physiology and Biochemistry, Faculty of Biology, St. Petersburg State University, 199034 St. Petersburg, Russia; le_r_ka@inbox.ru; 3 Department of Scientific Information, Russian Academy of Sciences Library, 199034 St. Petersburg, Russia

**Keywords:** acetylation, brown algae, cell wall, *Fucus*, LC-MS profiling, *Pelvetia*, phlorotannin fingerprint, physodes

## Abstract

Accumulation of biologically active metabolites is a specific feature of plant biochemistry, directing the use of plants in numerous applications in the pharmaceutical and food industries. Among these substances, the plethora of phenolic compounds has attracted particular interest among researchers. Here, we report on new findings in phlorotannin research, a large group of multifunctional phenolic substances, produced in brown algae. Comprehensive LC-MS profiling of three algal species allowed us to depict the complex pattern of this structurally diverse compound group across different tissues and subcellular compartments. We compiled more than 30 different phlorotannin series in one sample and used accurate mass spectrometry to assign tentative structures to the observed ions based on the confirmed sum formulas. From that, we found that acetylation, hydroxylation, and oxidation are likely to be the most common in vivo modifications to phlorotannins. Using an alternative data mining strategy to cope with extensive coelution and structural isomers, we quantitatively compared the intensity of different phlorotannin series in species, tissues, and subcellular compartments to learn more about their physiological functions. The structure and intra-thallus profiles of cell wall-bound phlorotannins were studied here for the first time. We suggest that one of the major dibenzodioxin-type phlorotannin series may exclusively target integration into the cell wall of fucoid algae.

## 1. Introduction

One of the most interesting biochemical features of brown algae is their ability to synthesize and accumulate specific phenolic compounds—phlorotannins. These substances represent a complex mixture of oligomers and polymers, formed by combining different numbers of phloroglucinol (1,3,5-trihydroxybenzene) molecules. The degree of polymerization (DP) varies broadly, so that the molecular weight of phlorotannins ranges from 126 Da of the monomer to 650 kDa [1]. Based on the nature of inter-monomer covalent bonds and the number of hydroxyl groups, phlorotannins are divided into different structural classes. The first class, characterized by ether inter-monomer linkages, includes fuhalols and phlorethols; the second class comprises fucols having only aryl-aryl linkages; phlorotannins of the third class, fucophlorethols, form branched structures based on both aryl and ether inter-monomer bonds; molecules of the fourth class, eckols and carmalols, contain dibenzodioxin linkages [2]. This linkage polymorphism together with positional isomerism gives rise to a great structural diversity of phlorotannins [3].

Phlorotannins have been found so far in all studied brown algae (Ochrophyta, Phaeophyceae), and their content in algal tissues varies from 0.5 to 30% dry weight [4,5,6]. This characteristic is species-specific with the maximum phlorotannin level being reported from representatives of the orders Fucales and Dictyotales [1,7]. Besides inter-species differences, phlorotannin content in algal cells correlates with water salinity, nutrient availability, plant size, and developmental stage [1,8,9,10,11].

Phlorotannins are usually concentrated in epidermal and outer cortical cells of brown algal thalli. Algal cells contain two sub-cellular fractions of phlorotannins: intracellular and cell wall (CW)-bound ones. Water-soluble intracellular phlorotannins are sequestered in physodes, specific membrane-bound organelles [12]. Numerous physodes occupy a considerable part of the cytoplasm in brown algal cells [1,12]. CW-bound phlorotannins are integral structural components of the brown algal cell wall matrix, where they are covalently cross-linked to alginates [13,14,15]. The content of CW-bound phlorotannins in algal cells is approximately ten times lower than that of the intracellular [16] and the molecular composition of this sub-cellular fraction of phlorotannins is virtually unstudied so far. Moreover, there is still very little information available about the biosynthesis of phlorotannins from both fractions in brown algal cells. The phlorotannin monomer, phloroglucinol, is synthesized from malonyl-CoA through the acetate-malonate (polyketide) pathway by polyketide synthase type III [17]. Further, the immense diversity of phlorotannin molecules is considered a result of successive phloroglucinol polymerization reactions, catalyzed by vanadium-dependent haloperoxidases, but detailed mechanisms are still unclear [14,18,19].

Apparently, phlorotannins perform multiple physiological functions in the cells of brown algae [20]. Due to their toxicity and antibiotic effects, they provide chemical defense against infection, biofouling and grazing [21,22,23]. Like many other phenolic compounds, phlorotannins have antioxidant activity and thus can protect algal cells against UV radiation and oxidative stress [24,25]. They are indispensable structural components of the brown algal cell wall [15] and adhesive material [19,26]. Phlorotannins contribute to wound healing [23] and to the key stages of brown algal embryogenesis [18,27,28,29]. Notably, recent data imply that different physiological functions might be attributed not just to the whole pool of cellular phlorotannins, but to specific phlorotannin molecules [20]. Thus, antioxidative activity and efficiency of chemical protection depend on phlorotannin chemical structure and DP [24,30,31]. Moreover, it was shown that cells of different thallus zones of brown algae contain different amounts of intracellular phlorotannins [5,6,32,33]. Apparently, this data reflects physiological specialization of cells in macroalgae with complex, morphologically and physiologically differentiated thalli, like those of Fucales and Laminariales. Unfortunately, most of these studies comprised methods that only allowed the determination of the intracellular total phenolic content s in the brown algal cells. This approach cannot reveal the structural diversity of individual phlorotannin molecules inside the physodes and gives no information about CW-bound phlorotannins. We suggest that cells of different thallus zones may contain not only different amounts, but also a different spectrum of phlorotannins in both intracellular and CW-bound fractions. Consequently, different physiological functions may be attributed to specific groups of phlorotannin molecules.

Therefore in this study, we used LC-MS analysis to obtain a comprehensive picture of molecular profiles of two subcellular phlorotannin fractions (intracellular and CW-bound) in four thallus zones of three species of the family Fucaceae, differing in size, lifespan and ecology. Based on the obtained results, we confirm the concept of distinct molecular functions of phlorotannins in dependence on their chemical structure.

## 2. Results

### 2.1. General Description of Phlorotannin Chromatograms and Molecular Profiles

**Figure 1:**
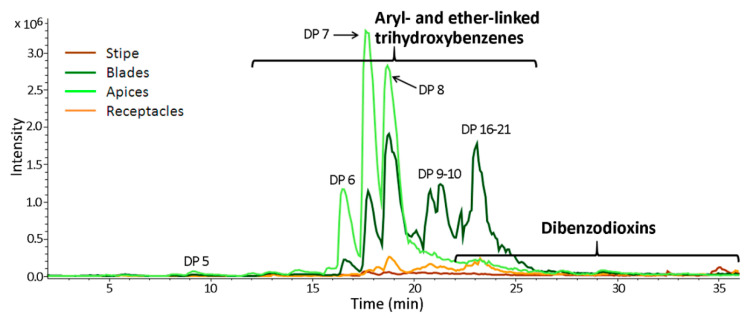
Typical base peak chromatograms of the intracellular phlorotannin extracts of four thallus zones of *F. serratus*. **DP**—degree of polymerization. The corresponding thallus zone is indicated by different coloring according to the legend on the left side of the graph. Enhanced coelution is observed with ascending degree of polymerization. Dioxin structures are more strongly retained on the reverse phase column.

Since reversed-phase interaction separates analytes basically in response to their polarity, the phlorotannins of the same chemical basic structure were separated corresponding to their degree of polymerization. Typical chromatograms obtained from different thallus zone samples are presented in Figure 1 on the example of *Fucus serratus* extracts (for more details, including representative replicates of all samples refer to Supplementary Materials, Figures S1–S3). A separation was only achieved for low molecular weight (LMW) phlorotannins, up to DP 10 (e.g., MW 1242.2 g mol^−1^) eluting between 5 and 22 min (Figure S2). This is consistent with previous HPLC-based studies of these compounds, showing that separation of higher molecular weight (HMW) phlorotannins by reversed-phase interactions is not feasible for two reasons: (1) the large, increasing number of isomers, and (2) the descending difference in polarity between two molecules with ascending degree of polymerization. Both result in extensive coelution and the observation of either broad peaks or unresolved “humps” (e.g., [34,35]). Maximal DP detected for the most abundant phlorotannin series are presented in Supplementary Materials (Table S1).

Figure 1 illustrates that although the basic structure of the particular phlorotannin also influences retention, coelution appears over a wide range of retention time (compare also Figures S2 and S3 given in the Supplementary Materials).

**Figure 2:**
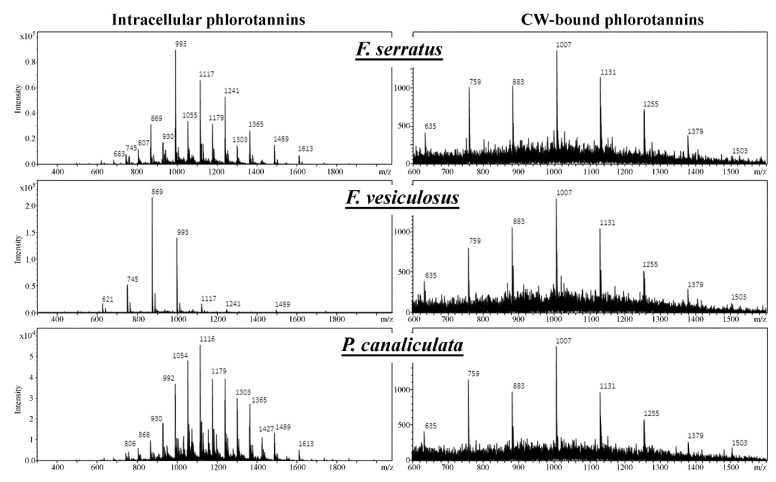
General *m/z* patterns in samples of intracellular and cell wall (CW)-bound phlorotannins from three species of Fucaceae: *F. serratus*, *Fucus vesiculosus*, and *Pelvetia canaliculata*.

The appearance of the mass spectra averaged over the RT corresponding to the HMW phlorotannin peaks, the unresolved “hump”, is dominated by the polymeric nature of the target analytes having characteristic profiles of equidistant *m/z* clusters with 124 u difference (Figure 2). Thus, each group of phlorotannin molecules having the same basic structural unit but differing in the degree of polymerization, manifests as a specific *m/z* series with an increment corresponding to an additional phloroglucinol moiety (i.e., 124 u = C_6_H_4_O_3_, Δ*m/z* according to the charge of the molecule).

**Figure 3:**
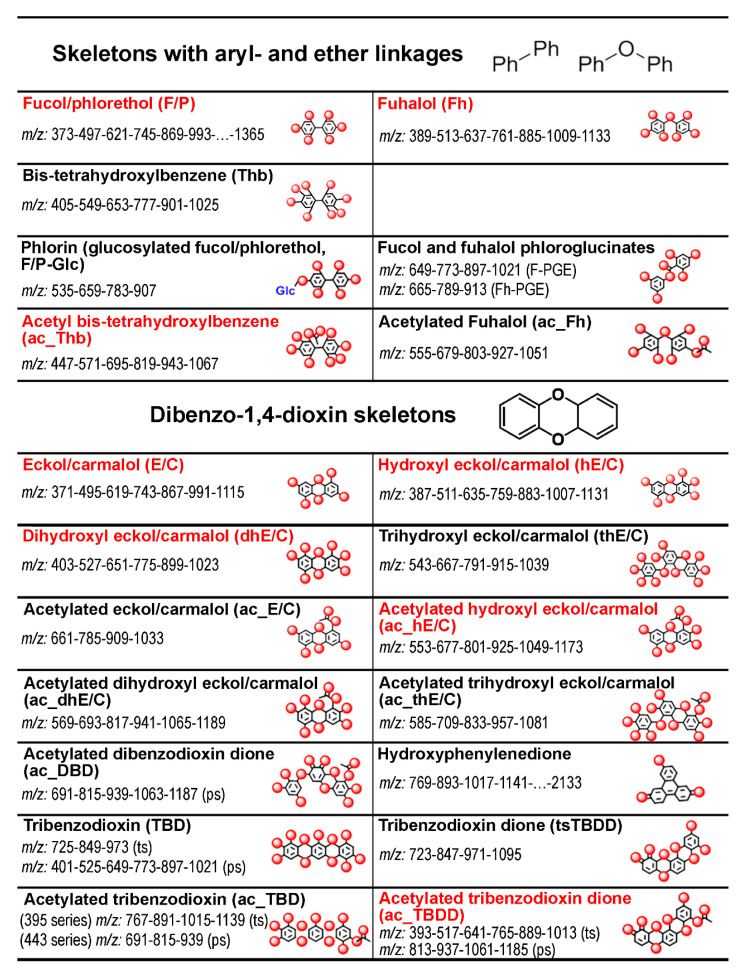
Twenty-one principal series (31 in total including the different hydroxylation grades, see Table S2) with exemplary structures of phlorotannin building blocks proposed for different phlorotannin series found in the three fucoid algae, and the corresponding *m/z* series. Each structure proposal is given a trivial name incorporating the proposed basic structure and an abbreviation to be used as a label in the following text and figures with **ac** (acetylated), **h** (hydroxylated), and the initials of the name of the basic structural unit. The proposed basic chemical structure is also exemplified in an icon-like structure drawing placed right from the series name, where all oxygen atoms are illustrated as red spheres while hydrogen atoms are not shown. All sum formulas of the listed series were confirmed by accurate mass analysis using abundant species at different charge states (Table S2). Red-colored font indicates that the series was quantified. **ts**, tetra-substituted and **ps**, penta-substituted phenol.

Comprehensive analysis of phlorotannin structural diversity in different species, physiologically differentiated thallus zones and subcellular compartments showed that the pool of these metabolites in algal cells is even more complex than suggested in previous studies. More than thirty *m/z*-based series of phlorotannin molecules were distinguished simultaneously in algal extracts (Figure S2; Table S2). Moreover, due to intrinsic isomerism of these compounds (e.g., fucols and phlorethols, F/P), each series likely refers to more than one phlorotannin structural class as suggested by the number of signals already detected for low DPs (Figure S2). Unfortunately, extensive coelution prevented separate quantitative assessment of the many isomers. For 21 of the distinguished phlorotannin series, we were able to develop at least one proposal for the tentative structure (Figure 3), deduced from the sum formulae considering the observed M–H^+^ and the elements C–H–O only and finally adopting recognized chemical modifications from the literature.

From Figure 3, it can be summarized that the basic structure of the phlorotannin series in these three species is diversified mainly based on four structural principles, namely hydroxylation, advanced dioxin linkages, acetylation and oxidation, while tentative glycosylation and phloroglucinate esterification may play only a very minor role. For the proposed acetylation, artefact formation during extraction with ethyl acetate was ruled out after comparing the intensity of these series between extraction protocols with and without the use of ethyl acetate (data not shown); it is also unlikely that a corresponding reaction would take place at all under the chosen conditions [36]. The acetylated phlorotannin series usually had a lower response, compared to the non-acetylated ones (on average, 25% of the total pool of intracellular phlorotannins). From 21 series with suggested tentative structures, one third were actually proposed to be acetylated analogues of other phlorotannin species where dibenzodioxin species seemed more prone to this modification.

The chromatograms of CW-bound phlorotannins contained no chromatographically separated peaks (Figure S3) but when averaging the mass spectrum over the corresponding chromatogram region, a single phlorotannin series could be observed. MS analysis of this subcellular fraction revealed this to be a dibenzodioxin-type phlorotannin series (hE/C) of LMW molecules with DP 4–11 (Figure 2).

In our analysis, the response of the CW-bound phlorotannin *m/z* series demonstrated a much lower intensity than that of intracellular phenols and was lower by more than a factor of 50. As to what extent this might be a consequence of incomplete hydrolysis and release of the phlorotannins during alkaline extraction cannot be concluded from our data. However, although additional, less abundant series in the CW-bound fraction would hardly be detected given the low absolute intensity of the single observed series, our data still clearly suggests that both phlorotannin fractions have a rather opposing composition with respect to the basic structural units, i.e., aryl- and ether- for the intracellular vs. dibenzodioxin-linkages for the CW-bound phlorotannins.

### 2.2. Total Phlorotannin Content

**Figure 4:**
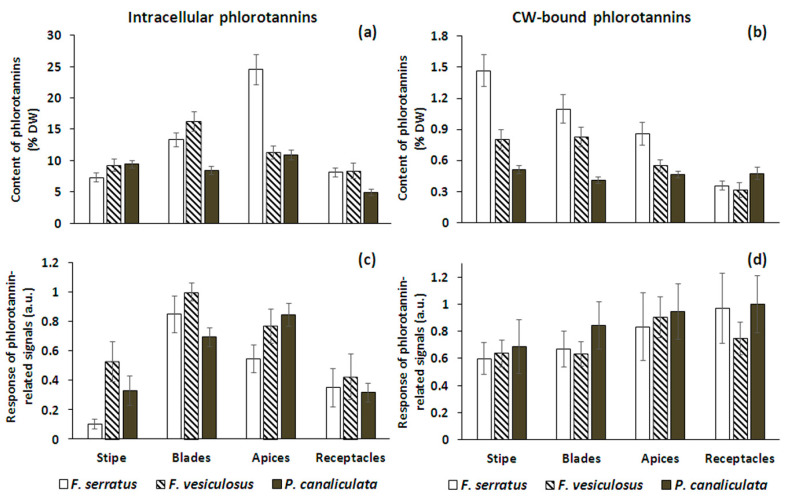
Total content of intracellular (**a**,**c**) and cell wall (CW)-bound (**b**,**d**) phlorotannins in different thallus zones of three species of Fucales, measured by the Folin–Ciocalteu method (**a**,**b**) and estimated as summed intensities of the averaged phlorotannin-related signals after LC-MS analysis (**c**,**d**); **a.u**., arbitrary units (averaged number of ions per scan, normalized to the maximum value). Bars represent the mean ± SD (*n* = 12 for **a**, **b** and 5 for **c**, **d**).

We compared two strategies to estimate the phlorotannin content in the analyzed samples—one, quantification based on the Folin–Ciocalteu method, and the other, based on cumulative intensities of the averaged phlorotannin-related signals after LC-MS analysis (Figure 4).

The total amount of intracellular phenolic compounds in the algal tissues varied from ~5 (receptacles of *P. canaliculata*) to ~25 (apices of *F. serratus*) % DW (Figure 4a). Both *Fucus* species demonstrated the specific intra-thallus profiles of phlorotannin distribution with maximum in the blades and apices and minimum in the receptacles and stipe; the distribution of polyphenols along the thallus of *P. canaliculata* was more even. For this alga, the most pronounced difference was observed between the vegetative and reproductive tissues—the content of intracellular phlorotannins in the receptacles was approximately two times less than in the other thallus zones (Figure 4a).

The total content of CW-bound phlorotannins in all studied species was an order of magnitude lower than the content of intracellular phlorotannins, varying from 0.3 (receptacles of *F. vesiculosus*) to 1.5 (stipe of *F. serratus*) % DW (Figure 4b). Notably, in all three species the distribution profiles of this phlorotannin fraction were different from those of intracellular phlorotannins. In both *Fucus* species, CW-bound phlorotannins mostly accumulated in the stipe and blades with a minimum content in the receptacles. *P. canaliculata* again demonstrated a more even phlorotannin distribution and, having a generally lower CW-bound phlorotannin concentration than the two *Fucus* species, contained a relatively high amount of these substances in the receptacles. Thus, for both *Fucus* species, a proportion of CW-bound phlorotannins in the overall pool of cell phenolic compounds was maximal in the stipe, and for *Pelvetia*, in the receptacles (Figure 4a,b).

As LC-MS analysis allowed us to only detect molecules with a DP up to 38, the abundance of phlorotannins, calculated as summed intensities of the phlorotannin-related signals (Figure 4c,d), may rather be a proxy of the LMW portion of the total phlorotannin pool. In the case of intracellular phlorotannins of *P. canaliculata* and, especially, *F. vesiculosus*, these data were generally consistent with the results of absolute quantification (higher content in blades and apices, and lower in stipe and receptacles) (Figure 4a,c). The only considerable difference refers to the stipe and apices of the third species, *F. serratus*, which showed relatively low intensity of intracellular phlorotannin-related signals, in comparison to the parallel data of total phenolic content (Figure 4a,c). Thus, we suggest that stipe and apices of this alga are enriched with phlorotannin molecules, inaccessible for LC-ESI-MS (>DP38). On the contrary, the profile of the LC-MS-detectable portion of CW-bound differed from that of total phlorotannins in all three species (Figure 4b vs. Figure 4d). The maximum response of these molecules appeared in receptacle and apex samples (Figure 4d). In conclusion however, both methods provide rather complementary information and cannot replace each other in studies using phlorotannin concentration for their conclusions.

### 2.3. Specificity of the Phlorotannin Profiles of Different Algal Species, Thallus Zones and Subcellular Fractions

We used a three-way ANOVA to assess the significance of differences in the relative content of particular groups of intracellular and CW-bound phlorotannins (Table 1).

Our results show that the molecular profile of intracellular phlorotannins was significantly different with respect to all factors included in the analysis, i.e., thallus zone, basic phlorotannin structural unit (series), and degree of polymerization. Significant factor interactions further indicate that each thallus zone of the alga possesses a specific intracellular molecular profile with respect to both phlorotannin series and DP (Table 1). The algal species was not used as a variable here to keep the ANOVA design concise; the inter-species difference in intracellular phlorotannin profiles is clearly illustrated in Figure 2 and is consistent with the literature [35].

**Figure 5:**
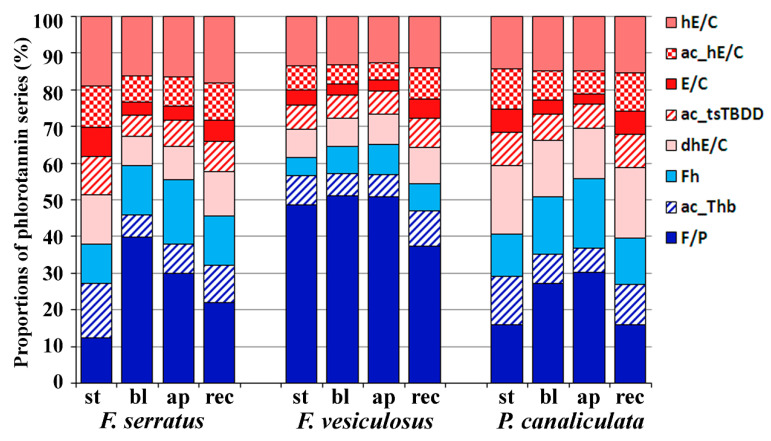
Relative contribution of eight major phlorotannin series to the pool of intracellular phlorotannins in different thallus zones of three Fucaceae species. **st**, stipe; **bl**, blades; **ap**, apices; **rec**, receptacles. Aryl- and ether-linked phlorotannins are in blue, dibenzodioxins are in red; uniform filling is used for free forms of phlorotannins, pattern filling is used for acetylated molecules.

Phenolic molecules belonging to different series contributed unequally in relation to the total pool of phlorotannins detected in the three brown algal species (Figure 5). *F. vesiculosus* featured the highest abundance of F/P series, which dominated the profiles of all its thallus zones. The other two species contained relatively higher levels of Fh, dhE/C and ac_hE/C series and demonstrated considerable variation in intra-thallus profiles (please refer to Figure 3 for the series abbreviations). Extracts of the stipe and receptacles had a generally lower overall concentration of phlorotannins (Figure 4) and contained the highest proportion of dibenzodioxin-type series (up to 62% of total amount in the stipes of *F. serratus* and receptacles of *P. canaliculata*), and the lowest of F/P series (Figure 5). Apices were relatively rich in fuhalols (the most pronounced in *Pelvetia*, the least in *F. vesiculosus*). Compared to the other species, *F. vesiculosus* demonstrated minimal inter-zone differences in phlorotannin series distribution.

Several features of phlorotannin profiles were common for all three species. (1) The series with the highest contribution were F/P, Fh, hE/C and dhE/C. (2) E/C series everywhere was the least abundant. (3) The proportion of acetylated phlorotannin series was maximal in stipes and receptacles (Figure 5).

**Figure 6:**
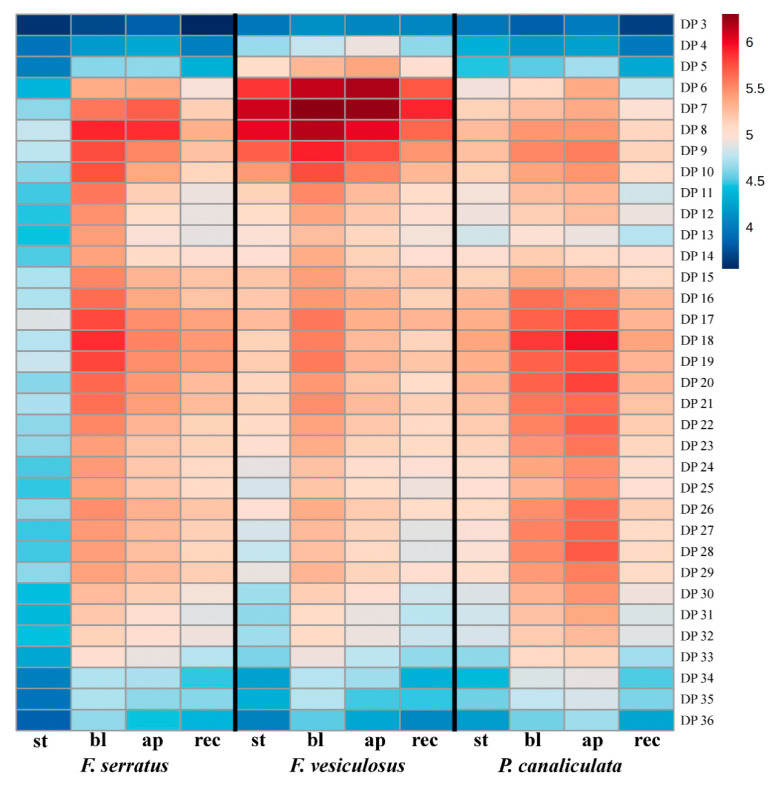
Relative contribution of molecules with DP 3–36 into the total pool of intracellular phlorotannins of different thallus zones of three Fucaceae. Mean values of five samples are presented on a log_10_ scale; **st**, stipe; **bl**, blades; **ap**, apices; **rec**, receptacles.

A comparison of the DP profiles of intracellular phlorotannins also revealed considerable differences between algal species and thallus zones (Table 1; Figure 1 and Figure 6). The relative molecular weight of detected phlorotannins decreased in the order: *P. canaliculata*, *F. serratus*, *F. vesiculosus*. Thus, *P. canaliculata* accumulated phlorotannins of DP 17-33, and *F. vesiculosus* contained a relatively high amount of phlorotannins with DP 4–10 (Figure 6). Besides inter-species variation, there was also a difference between the DP profiles of thallus zones. In both *Fucus* species, the apices contained less phlorotannins with DP 9–36 than blades, whereas the content of molecules with lower MW was the same, or higher. In all algae, stipe and receptacles had very similar DP profiles (*r* = 0.96–0.97), resembling those of the blades of the corresponding species (*r* = 0.86–0.98); though with lower total phlorotannin response (Figure 6). In all the analyzed samples, phenos with DP 2 and 37–38 had the lowest level (data not shown). Among studied species, *F. serratus* demonstrated the most pronounced difference in the zone-specific series and DP profiles of intracellular phlorotannins (Figure 1, Figure 5 and Figure 6).

**Figure 7:**
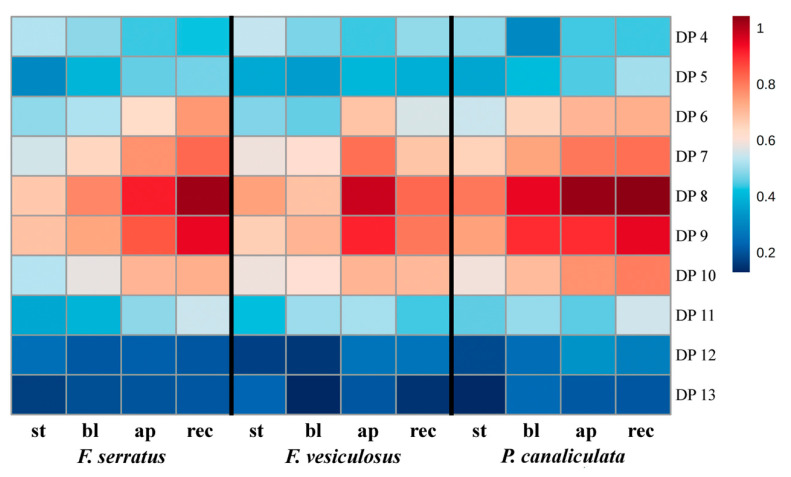
Relative contribution of molecules of hE/C series with DP 4–13 into the total pool of cell wall-bound phlorotannins of different thallus zones of three Fucaceae. Mean values of five samples are presented on a log_10_ scale; **st**, stipe; **bl**, blades; **ap**, apices; **rec**, receptacles.

A striking difference was revealed between the profiles of phlorotannins isolated from two subcellular compartments of fucoid algae. Compared to the intracellular phlorotannins, CW-bound molecules demonstrated much less diversity. This subcellular fraction contained only relatively LMW (DP 4–13) phlorotannins, belonging to hE/C series (Figure 2 and Figure 3), and all thallus zones of three algal species had the same DP profile with dominating DP 8–9 (Figure 7). This is confirmed by ANOVA results showing no significant interaction between species, thallus zone and phlorotannin DP (Table 1). Thus, molecules with DP 8–9 contributed the most to the difference in total response of LC-MS-detectable phlorotannins between algal thallus zones (Figure 4d and Figure 7). Such “deficient” molecular profiles of CW-bound phlorotannins are definitely not an artifact imposed by alkaline hydrolysis (see Materials and Methods), as all phlorotannin series were still detected in a sample of intracellular phlorotannins treated with NaOH according to the same protocol (data not shown).

## 3. Discussion

The occurrence of the major phlorotannin series (F/P) in representatives of the family Fucaceae is consistent with literature data. Fucols and/or (fuco)phlorethols with DP 6–23 were found in both *F. vesiculosus* and *F. serratus* [31,37], as well as in *P. canaliculata* [35]. In extracts of *F. spiralis*, both fucols and fucophlorethols were detected and confirmed by nuclear magnetic resonance (NMR) analysis [38]. Data implies that the simultaneous occurrence of these two structural classes of phlorotannins may be common for fucoid algae. Unlike the F/P series, fuhalols were previously mostly reported from the family Sargassaceae (genera *Sargassum*, *Cystoseira*, *Bifurcaria*) [39,40,41]. We found only one study where LMW (DP 4–7) fuhalols and hydroxyfuhalols were detected in *F. vesiculosus* [42]. Similarly, phlorotannins with dibenzodioxin skeletons (eckols and carmalols) are supposed to be characteristic of Laminariales (genera *Ecklonia*, *Eisenia*, *Carpophyllum*) [43,44,45]. Nevertheless, our results imply that fuhalols, as well as eckols and their derivatives (series hE/C, dhE/C, ac_hE/C), occur in fucoid extracts with responses comparable to that of the F/P series (Figure 5). The most probable explanation of this discrepancy is that typically only the middle zone of algal thalli (blades) was analyzed, and phlorotannin extracts were subjected to extensive multi-step purification, leading to progressive dilution (e.g., [46]). According to our data, both fuhalols and dibenzodioxin-type compounds were more prominent, not in the blades, but in other zones of algal thalli. For example, the hE/C series dominated the phlorotannin profile of *F. serratus* stipe, and Fh contributed most to the profile of *P. canaliculata* apices (Figure 5). Among dibenzodioxin phlorotannins detected in our study, the hE/C series is of particular interest, because it was the only one found in both intracellular and CW-bound fractions (Figure 2). Molecules with the same masses (DP 2–8) were previously reported only from *Sargassum fusiforme* [41] and, up to now, were never seen in Fucaceae.

Detection and tentative identification of several series of naturally acetylated phlorotannins merits special consideration because such molecules have not yet been found and are reported here for the first time (Figure 3). Possibly, the majority of phlorotannin studies focused on particular molecules (usually, dominating ones) and thus did not consider the whole profile; moreover, acetylation was often used as derivatization for studied phlorotannins [45]. Similar reasons hampered the detection of naturally acetylated lignin in higher plants, which, as known now, may contribute up to 50% of lignin units in several species [47,48]. Considering possible biological rationales of this occurrence, we may suggest that acetylation is a variant of conjugation of phloroglucinol units on certain metabolic (e.g., storage or transport) or physiological (e.g., antimicrobial defense) purposes [49]. The conjugation hypothesis was further supported by the presence of additional series such as tentatively identified phloroglucinate esters and glycosylated species (Figure 3). However, additional structural analysis, such as NMR, will be pursued to confirm these tentative findings and develop a comprehensive idea of their physiological functions in algae.

The total content of intracellular and CW-bound phlorotannins in algal thalli (Figure 4a) corresponds well with the literature (5–14 and 0.8–2.3% DW, respectively), noting that this parameter varies considerably depending on the season and geographical location [16,32,50,51]. The most complicated phlorotannin profile with relatively high abundance of HMW species and a high proportion of dibenzodioxins (dhE/C, in particular) was detected in extracts of *P. canaliculata* (Figure 5 and Figure 6; Figures S1 and S3). Among the studied species, *Pelvetia* clearly stands apart due to both taxonomic and eco-physiological characteristics (Materials and Methods, Table 2). This is a miniature annual plant, inhabiting very harsh environments, where it is frequently subjected to desiccation, light and temperature stress. Steevensz et al. [35] suggested that the complex phlorotannin DP profile might reflect adaptation of this species to high-intertidal conditions. Moreover, *P. canaliculata* permanently hosts an endophytic fungus, thus gaining some lichen features [52]. As eckols exhibit considerable fungicidal activity [53,54], we suggest that relatively high levels of these phlorotannins in *P. canaliculata* (Figure 5) may help to control the fungus infection. Polyphenols are known to be involved in the interactions between vascular plants and symbiotic fungi; in particular, mycorrhizal infection leads to changes in polyphenolic profiles [55,56]. Sanders et al. [57] showed that the brown alga *Petroderma maculiforme*, occurring in both free-living and lichenized state, continued producing high abundance of physodes under the nutritional burden of supporting a fungal symbiont. Together with our results, this data suggests that certain phlorotannins (presumably, dhE/C series) may contribute to alga–fungus interactions.

Studies on the structural complexity of phlorotannin profiles and revealing the biological rationale underneath have become the most intriguing task of phlorotannin research. From this perspective, comparing the profiles of different tissues and cell compartments may potentially be more informative than inter-species comparisons. Variation of intracellular phlorotannin content in different thallus zones of fucoid algae was shown in previous studies. Though there are some inconsistencies in this data, most authors reported relatively low amounts of phlorotannins in the receptacles and a maximum in blades or apices (e.g., [32,58]), which corresponds well with our results (Figure 4a). In our study, we elaborate on this data by comparing not only total amounts of phenolic compounds, but also their molecular profiles (relative proportions of different phlorotannin series and DP (Figure 5 and Figure 6)). There is multiple evidence that biologically relevant phlorotannin characteristics depend on the size of the molecules. Thus, antioxidant properties, conferring protection against UV radiation, are mostly attributed to the molecules with lower DP [3,30,59]. Given these observations, the relatively high proportion of molecules with DP 5–8 in the apices of both *Fucus* species, compared to their blades (Figure 6), seems logical. Apices of these algae contain meristematic cells that need effective UV-protection because of the risk of DNA damage in frequently dividing cells [60]. Moreover, during the tides, apices are exposed to UV longer than blades because submerged thalli have an upright posture in the water, so that apices emerge first when the tide is falling, and submerge last, when the tide is rising. This is especially relevant for the low-intertidal/subtidal species, *F. serratus*, whose blades typically do not emerge at all during neap tides, and only apices are systematically exposed to air. In agreement, *F. serratus* demonstrated the most prominent difference between phlorotannin DP profiles of apices and blades (Figure 1 and Figure 6). A relatively high proportion of fuhalols in the apex profiles of all studied species is another feature, which potentially may enhance antioxidant properties of the corresponding phlorotannin pool (Figure 5). Fuhalols contain hydroxyl groups in the ortho/para-position, which was reported as one of the key factors conferring effective radical scavenging in phenolic compounds [55,61]. According to our data, tentative acetylation was also enhanced with the hydroxylation grade of such series with an expected higher reactivity.

Receptacles of all three species as well as stipes of *F. serratus* and *Pelvetia* had a particularly high proportion of dibenzodioxin phlorotannins. Surprisingly, *F. serratus* and *Pelvetia* stipes and receptacles possessed very similar profiles. Moreover, both zones showed more similarity with the corresponding zone of the other species, rather than with the other zones of the same alga (Figure 5). Dibenzodioxin phlorotannins are known for being the most toxic and exhibiting the strongest antimicrobial and anti-herbivory activity [23,53,54,62]. Thus, enrichment of the pool of intracellular phlorotannins with these compounds may enhance chemical protection of algal tissues. The optimal defense theory, predicting protective metabolite allocation in plants, claims maximum protection of those organs or tissues, which are the most crucial for whole plant survival and reproduction [32], i.e., to the receptacles as reproductive structures. However, stipe fitness is no less important for benthic algae, conferring their hold to the substratum and residence in the fucoid belt community, where reproduction success is maximal; gametes released by separated floating alga have very little chance of fertilization [63].

To our knowledge, this is the first study investigating intra-thallus profiles and the structure of CW-bound phlorotannins. Remarkably, the distribution of CW-bound phenols along algal thalli did not coincide with the intracellular pattern (Figure 4a,b). In both *Fucus* species, contribution of CW-bound molecules to the total pool of phlorotannins was maximal in the stipe (up to 17% in *F. serratus*), and *P. canaliculata* contained relatively more phlorotannins in the cell walls of the receptacles (up to 10%). As CW-bound phlorotannins are supposed to strengthen cell walls, such distribution must provide an additional toughness for stipe or receptacle cells. Therefore, phlorotannins may confer not only chemical but also mechanical protection of these thallus zones, particularly in the stipe for *F. serratus*, and the reproductive organs for *Pelvetia*. Such differential priority seems logical, because of all three species, *F. serratus* is the largest and most massive, while *Pelvetia* is the only annual species, preferably diverting important resources into reproduction.

Finally, the most exciting results appeared from LC-MS analysis of the CW-bound phlorotannin fraction. Unlike intracellular phlorotannins, CW-bound phenolics were represented by only one type of molecule, namely LMW dibenzodioxins of hE/C series (Figure 2). Moreover, different species and thallus zones, though demonstrating considerable variation in intracellular phlorotannin profiles, possessed the same DP profile in the cell wall (Figure 6 and Figure 7). Apparently, the origin of the CW-bound phlorotannin fraction in algal cells should be considered for explanation of this result. Physodes, which contain the pool of intracellular phlorotannins, derive from the endoplasmic reticulum and Golgi apparatus in the perinuclear region of the cell. Accordingly, phlorotannin monomers might be synthesized in the reticulum and then transferred to the Golgi for further processing [64]. Physodes gradually move to the periphery of the cytoplasm, where some of them secrete their contents into the apoplast via exocytosis [12]. After entering the apoplast, phlorotannins link to alginates, thus forming the CW-bound subcellular fraction of these phenols [13,14,15].

Based on appearance and histochemical data (toluidine blue staining), at least two types of physodes are distinguished in brown algal cells. As both types were observed simultaneously in the same cell, they were suggested to contain phenols of different structural features [64]. Toluidine blue metachromasia confirms that phenolic compounds are stained differently depending on their structure [65]. Relating this to our data, we suggest that phlorotannins may be synthesized and accumulated separately, one from another, based on different structural units; in particular, phlorotannins of the hE/C series might be located in specialized CW-targeted physodes. This series was detected in both intracellular and CW-bound phlorotannin fractions (Figure 1). Moreover, in all samples its relative contribution to the total pool of intracellular phlorotannins was very similar (on average, 17.5% for *F. serratus* and 14% for the other two species), compared to the other series (Figure 5). A slightly higher proportion of hE/C series in physodes of *F. serratus* may finally result in higher levels of CW-bound phlorotannins, measured in this species (Figure 4b). While we assume that a tight regulation of biosynthesis relates to the formation of the basic structural units, the high number of phlorotannin isomers observed in our data, rather suggests a somewhat chaotic formation during subsequent polymerization to macromolecules. The detailed mechanisms of phlorotannin incorporation into the cell wall are still unknown; these phenolic compounds are supposed to be activated by enzymatic oxidation and then cross-linked to alginates [14,15,19]. This may explain the selection of the dibenzodioxins as the CW-bound phlorotannin fraction, as they possess a higher reactivity compared to aryl- and ether-linked phenols and are more susceptible to oxidation.

## 4. Materials and Methods 

### 4.1. Plant Material Collection

**Figure 8:**
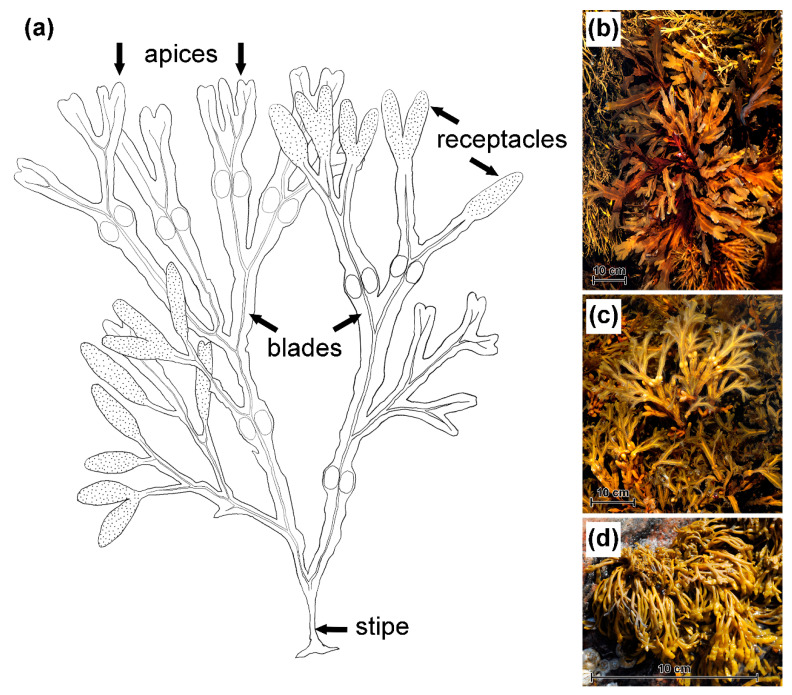
Objects of the study—three species of Fucaceae. (**a**) Schematic drawing of fucoid thallus (*F. vesiculosus*, as an example) illustrating the thallus zones used for phlorotannin analysis; (**b**) *F. serratus*; (**c**) *F. vesiculosus*; (**d**) *P. canaliculata*.

Samples from three species of Fucaceae (*Fucus serratus* L., *F. vesiculosus* L., and *Pelvetia canaliculata* (L.) Dcne and Thur.) were collected in the Keret Archipelago (Kandalaksha Bay, White Sea; 66°17’28.76” N 33°40’03.46” E) in August. Thalli with mature receptacles were collected from the typical habitats of each species (Table 2). To minimize possible variation of metabolite content in response to the tidal cycle, desiccation and warming during transportation, all plants were collected at high tide and directly transported to the laboratory in seawater. The algae were stored in seawater at 16 °C for no more than 1 h before sample preparation. All samples were taken from four thallus zones: stipe (lowest part, just above the holdfast), blades, apices (upper 3 mm for both *Fucus* species, and 2 mm for *P. canaliculata*) and mature receptacles (Figure 8).

### 4.2. Total Phlorotannin Content

Extraction of intracellular and CW-bound phlorotannins was performed according to [16] with modifications. Briefly, 20 mg (fresh weight) plant material was poured with acetone:water (70:30, *v/v*) mixture, ground with mortar and pestle and left soaking in 1 mL aqueous acetone for one hour to extract intracellular phenolics. Then, the extract was centrifuged (5000× *g*, 10 min), the supernatant was transferred into another tube, and the pellet was re-extracted with another 1 mL of aqueous acetone. The supernatants of five extraction rounds were combined. The CW-bound phlorotannin fraction was extracted from the precipitate of the remaining algal material after the extraction of intracellular phlorotannins. The precipitate was resuspended in 0.5 mL of 1 M aqueous NaOH solution (80 °C) and then incubated for 2.5 h at room temperature with continuous shaking (750 rpm). After centrifugation (5000× *g*, 10 min), the supernatant was transferred to another tube. The alkaline extraction was repeated three times. The combined supernatants were neutralized with concentrated HCl to pH 6.8–7.0.

A modification of the Folin–Ciocalteu micro-method was used to measure the total phenolic content in the samples [66]. Phloroglucinol (Sigma-Aldrich 79330) was used as the standard. The reaction mixture containing 0.3 mL of sample, 0.3 mL of Folin reagent and 2.4 mL of 5% (*w/v*) Na_2_CO_3_, was incubated for 20 min at 45 °C, and then the absorbance was measured at 750 nm using a SPEKOL 1300 spectrophotometer (Analytik Jena AG, Jena, Germany). The phlorotannin content was expressed as percentage of the dry weight.

### 4.3. HPLC-ESI-MS Phlorotannin Profiling

Acetone was evaporated from the combined aqueous acetone extracts of soluble phlorotannins in a speedvac (vacuum centrifuge concentrator plus, Eppendorf, Hamburg, Germany). The combined alkaline extracts of CW-bound phlorotannins were acidified to pH 2.8–3 with concentrated hydrochloric acid. Then, both types of extracts were defatted, partitioning three times against dichloromethane (1:1, *v/v*), and phlorotannins were extracted by five successive portions of ethyl acetate (1:1, *v/v*). Ethyl acetate extracts were dried in a speedvac and resuspended in 0.15 mL water for subsequent HPLC-MS analysis.

Samples (50 μL) were injected to an Agilent 1100 HPLC system (Agilent, Waldbronn, Germany) equipped with a reversed-phase column Gemini C18, 5 µm, 110 Å, 150 mm × 2 mm (Phenomenex, Aschaffenburg, Germany). Gradient elution at a flow rate of 0.4 mL/min was employed to separate the sample components using 0.1 % formic acid in water (B) and acetonitrile (A) as eluents using the following program (time in min/%B): 0.0/100, 10.0/100, 20.0/70, 30.0/70, 40.0/0, 50.0/0. MS data was acquired on a Bruker Esquire 3000 Plus ESI ion trap mass spectrometer (Bruker Daltonics, Bremen, Germany) in negative ion mode at a nebulizer pressure of 40 psi, dry gas flow at 9 L/min and dry gas temperature of 365 °C with *m/z* 1000 as the target mass.

Data evaluation was based on cumulative peak intensity in averaged mass spectra as detailed in the Supplementary Materials (Figures S1–S4).

### 4.4. Accurate Mass Analysis of Phlorotannin-Related Signals

Accurate mass analysis was used to confirm the sum formulas of our tentative structural assignments on the example of the species detected in samples of *F. vesiculosus* blades. For this, 50 µL sample was injected to an Ultimate 3000 UHPLC system (ThermoScientific, Waltham, MA, USA). Separation was accomplished at 30 °C on a Gemini 5 µ C18 110 Å, 150 mm × 2 mm column with a 2 mm guard column of the same material (Phenomenex Ltd., Aschaffenburg, Germany). The following gradient with 0.1% formic acid in ACN (A) and 0.1% aqueous formic acid (B) as eluents was selected and run at a flow rate of 400 µL min^−1^: 100% B (0–10 min), to 70% B in 10 min held for 10 min, to 0% B in 10 min held for 10 min, and equilibration with 0% B to 100% B in 5 min held for 10 min. The outlet was connected to a Bruker Daltonik (Bremen, Germany) Impact II ESI-QqTOF-MS (Quadrupole-Time-Of-Flight) in negative ion mode controlled by Bruker Daltonics Hystar 3.2 SR4 and otofControl 4.0.21.1960. The ESI source was set to 4500 V capillary voltage, 3 bar nebulizer pressure, 10 L/min dry gas flow, 250 °C dry gas temperature (both nitrogen) and the following ion sampling parameters: Funnel 1/Funnel 2/Hexapole/Collision RF = 150/200/50/800 Vpp; Ion and Collision Energy = 4 and 7 eV; Transfer Time and Pre Pulse Storage = 100 and 5 µs.

Mass spectra were acquired between *m/z* = 50–3000 and analyzed with Bruker Data Analysis 4.2 SR1. All proposed formulas were confirmed within an expected deviation < 5 ppm in comparison to the theoretical (calculated) masses from the proposed structure (Table S2) for all signals featuring intensities within the dynamic range.

### 4.5. Data Analysis

Measurements were performed with five (LC-MS analysis) to twelve (total phenolic content) replicates. Analysis of variance (ANOVA) was carried out using Statistica 7.1 (StatSoft Inc., Tulsa, OK, USA). We used a three-way factorial ANOVA design, taking thallus zone, phlorotannin series and phlorotannin DP as variables for analysis of intracellular phlorotannin profiles, and species, thallus zone and phlorotannin DP as variables for analysis of CW-bound phlorotannin profiles. Excel 2013 (Microsoft, Redmond, WA, USA) and MetaboAnalyst 4.0 Web application were used for data processing, normalization procedures and heatmap construction [67]. All values are expressed as means and standard deviations.

## 5. Conclusions

Our results present a comprehensive, multi-layered “snapshot” of phlorotannin profiles in cells of fucoid algae. More than thirty series derived from phlorotannin basic structural units were detected in extracts from different tissues of *F. serratus*, *F. vesiculosus* and *P. canaliculata.* Sum formulas were deduced from the accurate *m/z* of the molecular ions and used to assign tentative structures to these units, considering the literature data. Among those, several phlorotannin series (including naturally acetylated ones) are reported here for the first time. For evaluation of these complex data, we developed a semi-quantitative approach based on cumulative assessment of averaged mass spectra paying special attention to the broadly coeluting isomers and overlapping polymer series and charge states.

This data revealed both quantitative and qualitative differences in phlorotannin profiles on species, tissue and cell level in the three studied algae; we suggest that this variation reflects distinct physiological features of these metabolites. Thus, phlorotannins integrated into cell wall, comprise only dibenzodioxin-type molecules belonging to series hE/C. To our knowledge, this is the first example of a specific physiological function being clearly attributed to a particular group of phlorotannin molecules. Moreover, the repetitive profile of CW-bound phlorotannins across different species and thallus zones implies that this function might be very conservative in biochemical evolution of brown algae. Based on our results, further investigations can now be initiated to advance our findings and elaborate on the concept of distinct metabolic functions with respect to phlorotannin chemical structures.

## Figures and Tables

**Table 1:** Summary of ANOVA results for the quantitative data on the eight most abundant phlorotannin series illustrated in more detail in Figure 5, Figure 6 and Figure 7.

Variable	df	*F* Ratio	*P*
**Intracellular Phlorotannins**			
Thallus zone	3	181.13	<0.001
Phlorotannin series	7	275.83	<0.001
Phlorotannin DP	35	69.34	<0.001
Thallus zone × Phlorotannin series	21	31.72	<0.001
Thallus zone × Phlorotannin DP	105	4.37	<0.001
Series × Phlorotannin DP	245	26.20	<0.001
Thallus zone × Phlorotannin series × Phlorotannin DP	735	1.76	<0.001
**CW-Bound Phlorotannins**			
Species	2	3.65	<0.001
Thallus zone	3	31.30	<0.001
Phlorotannin DP	9	79.32	<0.001
Species × Thallus zone	6	8.70	<0.001
Species × Phlorotannin DP	18	0.77	0.74
Thallus zone × Phlorotannin DP	27	2.77	<0.001
Species × Thallus zone × Phlorotannin DP	54	0.73	0.92

**Table 2:** Characteristics of the investigated three species of Fucaceae harvested on shores of the Kandalaksha Bay, White Sea.

Fucaceae Species	Size	Lifespan	Typical Habitat	Reproductive Period
*F. serratus*	0.8–1.2 m	perennial	Subtidal-low intertidal	August–September
*F. vesiculosus*	0.4–0.9 m	perennial	Mid-intertidal	July–September
*P. canaliculata*	0.05–0.12 m	annual	high intertidal, wave-exposed rocks	August

## Supplementary Materials

The following are available online at https://www.mdpi.com/2218-1989/10/9/369/s1. References [68,69,70,71,72] are cited in the supplementary materials. Figure S1: Zoom into the *m/z* range between the most abundant degrees of polymerization, 7 and 8, of intracellular phlorotannin samples, Figure S2: Exemplary extracted ion chromatograms of DP 3–8 of the fucol/fucophlorethol series (*m/z* 373-1241) in *F. vesiculosus* and *F. serratus* blades, Figure S3: Elution profile of intracellular (left) and cell wall-bound phlorotannins (right) across the samples, Figure S4: Schematic illustration of the data mining workflow, Table S1: Maximal degree of polymerization (DP) detected for the most abundant phlorotannin series, the fucol/phlorethols, in all species, zones and fractions, Table S2: Confirmation of the sum formulas corresponding to the structure proposals by accurate mass analysis.
