# Supplementary material for: Composition of Intracellular and Cell Wall-Bound Phlorotannin Fractions in Fucoid Algae Indicates Specific Functions of These Metabolites Dependent on the Chemical Structure
Source: Metabolites. 2020 Sep 11;10(9):369. doi: 10.3390/metabo10090369 (PMC7570113; doi:10.3390/metabo10090369)
Supplement: Supplementary file 1 [file metabolites-10-00369-s001.zip › Supplementary Materials 1_rev.docx]

Supplementary materials

Composition of intracellular and cell wall-bound phlorotannin fractions in fucoid algae indicates specific functions of these metabolites dependent on the chemical structure

Claudia Birkemeyer ^1,^*, Valeriya Lemesheva ^2^, Susan Billig ^1^ and Elena Tarakhovskaya ^2,3,^*

^1^ Faculty of Chemistry and Mineralogy, University of Leipzig, 04103 Leipzig, Germany

^2^ Department of Plant Physiology and Biochemistry, Faculty of Biology, St. Petersburg State University, 199034 St. Petersburg, Russia

^3^ Department of Scientific Information, Russian Academy of Sciences Library, 199034 St. Petersburg, Russia; elena.tarakhovskaya@gmail.com

***** Correspondence: birkemeyer@chemie.uni-leipzig.de (C.B.); elena.tarakhovskaya@gmail.com (E.T.)

1. Approaches for identification of multiple phlorotannin series in the algae

In a first approach, we manually searched for phlorotannin series in the LC-MS chromatograms by the expected 124 u-difference between the molecules of ascending degree of polymerization. Other series not matching this phlorotannin building block such as those with a difference of 14 u, were not further analyzed. The corresponding *m/z* series were filed and then only matched to a tentative, basic chemical structure; thus, these series are the most abundant ones and were subjected to quantification in the following. Chromatograms of all algal species and zones were further manually searched to find and identify the minimum (2-3 for the intracellular and 5 for the cell wall-bound phenolics) and maximum degree of polymerization (DP) independent of the specific series (Table S1); however, since the fucol/phlorethol series was the most abundant we found the highest DP from this series.

| **Species/fraction** | **stipe** | **blades** | **apices** | **receptacles** |
| --- | --- | --- | --- | --- |
| **Intracellular** |  |  |  |  |
| *F. serratus* | 13 (18) | 16 (25) [35] | 16 (26) [37] | 15 (23) [31] |
| *F. vesiculosus* | 15 (21) | 14 (32) | 13 (26) | 13 (22) |
| *P. canaliculata* | 14 (25) | 17 (28) [38] | 16 (29) [38] | 14 (23) [31] |
| **Cell wall-bound** |  |  |  |  |
| *F. serratus* | 10 | 10 | 11 | 10 |
| *F. vesiculosus* | 10 | 12 | 10 | 11 |
| *P. canaliculata* | 11 | 12 | 10 | 10 |
|  |  |  |  |  |
|  |  |  |  |  |
| Legend: singly, (doubly), [triply] charged. | | | | |

**Table S1.** Maximal degree of polymerization (DP) detected for the most abundant phlorotannin series, the fucol/phlorethols, in all species, zones and fractions. The maximal DP (38) is labeled in bold. Brackets indicate the charge state of the highest m/z observed as outlined in the table footnote.

Additional series were identified by targeted search according to a literature survey on phloroglucinol and derivatives in algae and higher plants as potential building blocks for phlorotannins in the investigated fucoids (e.g. phlorethin [68], phlorin [69], furyl derivatives, acylphloroglucinols [70,71], phloroglucinic acid [28]). If evidence for the corresponding species was found in our data, these minor series were also filed to support the discussion related to the tentatively identified major series (see above) but not quantified due to the usually low abundance of these series. Exemplary structural proposals for the series are presented in Figure 4 in the main manuscript. Finally, we assessed all detected peaks between the most abundant DPs 7 and 8 (Figure S1) and assigned most of them based on structural motifs of already identified series filling “the gaps” of postulated reaction schemes, e.g. hydroxylated, acetylated and oxidized forms of already identified basic structures. This way, only *m/z* 887 and 985 remained without structural assignment.

**Figure S1.** Zoom into the *m/z* range between the most abundant degrees of polymerization, 7 and 8, of intracellular phlorotannin samples. Mass spectra were averaged between retention times 4–27 min in chromatograms obtained from the intracellular phlorotannin fraction of blade samples. In the top spectrum obtained from a *F. vesiculosus* sample, quantified series are colored in black while the non-quantified series are colored in grey. In addition, the most abundant signals of the quantified series are labeled with the exemplary structure proposal of the basic structural unit (see also Fig. 4, main manuscript). For a detailed description of the figure, all signals above the detection limit potentially originating from phlorotannin series are listed in the boxes placed above the ion current profile. While the spectrum of *F. vesiculosus* (top) is dominated by singly charged species (black), doubly charged (brown) and triply charged (green) species emerge in the spectra of *F. serratus* (middle) and *P. canaliculata* (bottom).

Figure S1 summarizes all *m/z* values of potentially present series clearly illustrating the structural diversity within the phlorotannin profile of the analyzed species. According to the suggested structures for unknown series, we propose hydroxylation, acetylation and oxidation to be the main principles of phlorotannin modification in these algae. Though the use of electrospray ionization may result in electrochemical redox reactions of target compounds as artifacts from analysis [72], the fact that we analyzed the phlorotannins in the negative ion mode renders *in situ*, i.e. in-source oxidation as artifact of the employed method unlikely. Thus, indeed we found no hints that the ratios of oxidized and reduced (quinone and phenol) form changed between positive and negative ion modes (not shown).

2. Development of a data evaluation strategy for quantification of the complex phlorotannin patterns

Chromatographic baseline separation of the complex phlorotannin mixture appeared impossible for degrees of polymerization beyond 10 (Figure S2) preventing from the application of targeted standard methods based on chromatographic peak finding and integration for quantification.

**Figure S2.** Exemplary extracted ion chromatograms of DP 3-8 of the fucol/fucophlorethol series (*m/z* 373-1241) in *F. vesiculosus* and *F. serratus* blades. Elution profiles suggest a rapidly increasing number of structural isomers (>9) with higher degrees of polymerization, concluded from the increasing number of peaks for each particular *m/z* eluting at different retention times; for higher DPs, coelution becomes too strong to further observe this effect and the signals merge creating plateaus.

LC-RP columns separate by polar/nonpolar interactions with the analyte; the polarity of molecules with higher DPs is more similar than at lower DPs so that an increasing number of isomeric molecular species with identical *m/z* coelute at later retention times disabling accurate automated peak recognition. Consequently, we had to develop an alternative quantification protocol to estimate the relative content of different phlorotannins. For this, we anticipated quantification based on the averaged intensity of the mass-spectral signals.

We carefully considered the following **critical issues** to compare signals all over the LC-MS chromatogram range in a semi-quantitative approach:

1. When averaging over low-noise and high-noise regions simultaneously, smaller signals in the low noise region might go undetected because averaging the noise as well.
2. Different charge states may overlap creating identical *m/z* values (e.g. a doubly charged molecule twice the degree of polymerization overlaps with its singly charged counterpart).
3. Species of higher molecular mass have a higher abundance of isotopic companion peaks leading to a bias in favour of low molecular weight species if quantifying the monoisotopic peak only which on the other hand is advantageous to prevent from inaccurate quantification due to overlapping with species of multiplied DP and charge state.

In conclusion, the selection of regions in the chromatogram used for averaging the mass spectra needs great care avoiding any distortion of the obtained phlorotannin pattern. Therefore, we developed the following **procedure for evaluation** of our data.

1. We identified and defined *three different noise regions* in the chromatograms:

| 2-17 min | Low noise with distinct chromatographic peaks |
| --- | --- |
| 16/17-25/27 min | Enhanced noise, overlapping chromatographic peaks and charge states |
| 25/27-35/38 min | High noise, no chromatographic separation, low abundant target peaks |

1. Charge states started overlapping in the chromatogram after 20 min retention time. This effect was species-specific: while for *F. vesiculosus* singly charged fucols/phlorethols are most abundant, 2-fold and even 3-fold charged species were observed in *F. serratus* which further increased in *P. canaliculata.* To prevent from fundamentally wrong assignment, we identified and defined *four different charge-state regions*:

| 2-22 min | singly charged phlorotannins with aryl and ether linkages |
| --- | --- |
| 16/17-24 and 28-36 min | singly charged dibenzo-1,4-dioxins |
| 20-24 min | doubly charged species in *F. serratus* and *P. canaliculata* |
| 24-28 min | triply charged species |

Consequently, we finally considered five different chromatogram regions in total to produce average spectra from our data: 2-17 min, 16/17-20 min, 20-24 min, 24-28 min, and 28-36 min (Figure S3).

For creating mass lists from the averaged spectra, we used the “find”/“mass list” feature of Bruker Data analysis 4.2 and iterated the default settings to find the following optimized parameters: peak width (FWHM) (*m/z*) 0.1; S/N threshold 0.25; relative intensity threshold 0; absolute intensity threshold 0. We used the scripting option to automatically average mass spectra within each of the defined five retention time regions for all 144 chromatograms and export the data as .csv files.

All expected target *m/z* corresponding to each DP of the eight quantified series were calculated across the three detected charge states (-1–-3). For identical *m/z* in this list, the charge state was selected matching the corresponding chromatogram region (see above, table of charge states). At this point, it was impossible to prevent from discrimination of less abundant overlapping species of the same *m/z* (mostly the higher charged species, i.e. higher degree of polymerization), so that our data still might be slightly biased towards lower DPs. If this appearance should be somehow relevant, it would be the most for *F. serratus* concluding from the mass spectra illustrated in Figure 3 in the main manuscript where the twofold charged species feature a rather high relative abundance while for *P. canaliculata* already the higher charge state was considered.

**Figure S3.** Elution profile of intracellular (left) and cell wall-bound phlorotannins (right) across the samples. Color-coding of the base peak chromatograms for each species was adopted from Figure 2 in the main manuscript: brown – stipe, dark green – blades, light green – apices, and orange – receptacles. Dashed lines indicate the chromatogram regions that were averaged and separately evaluated; five for the intracellular fraction and one region for the cell wall-bound phlorotannins. The maximal intensity between the two phlorotannin fractions differed by a factor >10 with *F. vesiculosus* featuring the highest maximal intensity across the species. While for the intracellular phlorotannin fraction, the profile considerably differed between the algal species and thallus zones, the chromatograms of the cell wall-bound phlorotannins were rather similar.

The target lists were searched against the averaged mass spectra from this region per chromatogram to extract the corresponding monoisotopic intensities. The cumulative abundance of the phlorotannin target *m/z* annotated by series number, degree of polymerization and charge, was obtained as the sum of the corresponding intensities in the averaged mass spectra from the five different regions within a particular chromatogram.

For quantification, we further considered the isotopic abundance of the species to correct for the higher probability of heavier atoms with increasing DP. To avoid systematic errors from overlapping of mass peaks from different series, we calculated the total abundance from the intensity of the monoisotopic peak considering the following equation from regression of the isotopic abundance as function of the mass m for the C-H-O containing phlorotannin species:

Abundance as [%] of the monoisotopic peak = f(m) = 99.8999*e^0.000583*m^

Because quantification of the fuhalols was compromised by the isotopic abundance of the hydroxyl eckol/carmalol series >M+2 contributing to the abundance of the signals corresponding to fuhalol peaks, we corrected the signal intensity of all M-H^+^ of the fuhalol series by subtracting the contribution of the corresponding M+2 signal before calculating the cumulative abundance. For clarity and summary, Figure S4 illustrates the process of data mining in a simple scheme.

For cell wall-bound phlorotannins, we detected only the hydroxyl eckol/carmalol series, which eluted between Rt 16-24 min so that only one average spectrum was used for evaluation. Compared to their counterparts from soluble phlorotannins, these chromatograms had a very low abundance and the average spectra featured a median abundance of phlorotannin peaks of 423 compared to a median noise of 192. Though the detection of eight representatives (DP 4-13) of the whole series enhanced the robustness for quantification, these values are near the detection limit and exhibited a high variance. When we used normalization to the median intensity of all signals in the spectrum to reduce the influence of the noise, the standard deviation only slightly improved but the main benefit was the better correlation of the normalized signal intensity with the observed signal to noise ratio. However, considering the low overall abundance in comparison with the least difference between the abundance of different phlorotannin series in the intracellular fraction by factor 3, the fact that we did not observe other than the dibenzodioxin series in the cell wall extracts still implies that less abundant series at least *could* be present and further experiments need to confirm the real absence of other series. On the other hand, it still suggests that the profile of the cell wall-bound phlorotannins definitely shifts from the aryl- and ether-linked series towards dibenzodioxins.

**Figure S4.** Schematic illustration of the data mining workflow.

Comparisons with blank measurements finally confirmed the accuracy of the extracted signals originating from the cell wall-bound extracts. For quantification of phlorotannin series in figures comparing zones, species and DP in the manuscript, the sum of abundances of the corresponding phlorotannin target *m/z* annotated by series number, degree of polymerization and charge, was calculated within a chromatogram (over the corresponding regions) and presented as mean of five replicates.

As a last comment, the following issues shall be mentioned as method-intrinsic which cannot be corrected by data treatment without suitable standards:

1. Later eluting (mainly the higher) *m/z* may exhibit a biased, higher relative intensity due to the higher electrospray ionization efficiency at higher organic fraction.
2. Relative signal intensity may be further related to the selected target *m/z* 1000 during acquisition. In fact, blanks showed enhanced noise intensities around *m/z* 900-1100 (DP 8-9 for the singly charged, 17-19 for the doubly charged and 26-29 for the triply charged). However, independent analyses of *F. vesiculosus* samples on a different-type instrument during accurate mass analysis confirmed DP 6-8 to be the most abundant in this species.
3. High molecular weight species may have a biased lower relative intensity due to poorer desorption during electrospray ionization.
4. Different phlorotannin isomers may feature a different ionization efficiency.

These issues can be addressed by calibration with authentic standards, but such standards are not available yet. Consequently, we feel that the availability of calibration standards is of utmost importance to enhance the quality of quantitative assessment in future phlorotannin research employing mass spectrometric techniques. Meanwhile, until suitable standards will become available, a complementary use of gel permeation chromatography with triple detection to validate corresponding LC-MS data in similar future experiments is highly recommended.

References

1. Mariadoss, A.V.A.; Vinyagam, R.; Rajamanickam, V.; Sankaran, V.; Venkatesan, S.; David, E. Pharmacological aspects and potential use of *phloretin: a systemic review. Mini-Reviews in Medicinal Chemistry* **2019**, *19(13)*, 1060-1067. https://doi.org/10.2174/1389557519666190311154425
2. Louche, L.M.-M.; Luro, F.; Gaydou, E.M.; Lesage, J.-C. Phlorin screening in various citrus species and varieties. *J. Agric. Food Chem.* **2000**, *48(10)*, 4728–4733. https://doi.org/10.1021/jf991362x
3. Song, C.; Ring, L.; Hoffmann, T.; Huang, F.-C.; Slovin, J.; Schwab, W. Acylphloroglucinol biosynthesis in strawberry fruit. *Plant Physiol.* **2015**, *169*, 1656–1670. https://doi.org/10.1104/pp.15.00794
4. Na, M.K.; Jang, J.P.; Min. B.S.; Lee, S.J.; Lee, M.S.; Kim, B.Y.; Oha, W.K.; Ahn, J.S. Fatty acid synthase inhibitory activity of acylphloroglucinols isolated from *Dryopteris crassirhizoma*. *Bioorg. Med. Chem. Lett.* **2006**, *16*, 4738–4742. https://doi.org/10.1016/j.bmcl.2006.07.018
5. Abburi, R.; Kalkhof, S.; Oehme, R.; Kiontke, A.; Birkemeyer, C. Artifacts in amine analysis from anodic oxidation of organic solvents upon electrospray ionization for mass spectrometry. *Eur. J. Mass Spec.* **2012**, *18(3)*, 301–213. https://doi.org/10.1255/ejms

| 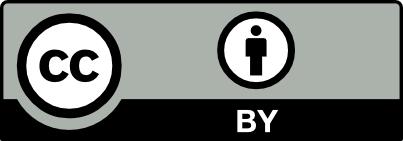 | © 2020 by the authors. Submitted for possible open access publication under the terms and conditions of the Creative Commons Attribution (CC BY) license (http://creativecommons.org/licenses/by/4.0/). |
| --- | --- |
